# Primary Hydatid Cyst of the Thyroid Gland

**DOI:** 10.1155/2011/713089

**Published:** 2011-08-01

**Authors:** Imen Azendour, Mohamed Boulaich, Ali Ayoubi, Abdelilah Oujilal, Leila Essakalli, Mohamed Kzadri

**Affiliations:** Département de ORL, Hôpital des Spécialités, Rabat, Morocco

## Abstract

Primary hydatid cyst of thyroid gland is an exceptional localization even in Morocco where echinococcal disease is endemic. A 23-year-old woman presented with multiples cystic lesions of the thyroid revealed by neck mass and dyspnea. She underwent a subtotal thyroidectomy. The diagnosis of hydatid cyst was made preoperatively and was confirmed by histological studies. Further investigation failed to identify any other evidence of systemic hydatidosis. The patient has remained asymptomatic for 24 months after surgery. The possibility of hydatid disease, though rare, should be always kept in mind, for patients with cystic lesions of the thyroid, because a needle aspiration biopsy is a potentially harmful procedure.

## 1. Introduction

Hydatid disease (HD) is a zoonotic infestation caused by Echinococcus granulosus and Echinococcus multilocularis. Thyroid gland is very rarely involved by this parasitic infection even in countries where the disease is endemic [1, 2]. Review of the literature has found less than 60 cases of isolated cystic echinococcosis of the thyroid gland since 1965 [1–4]. We report a case with primary hydatid cyst situated in the right lobe of the thyroid gland.

## 2. Case Report

**Figure 1:**
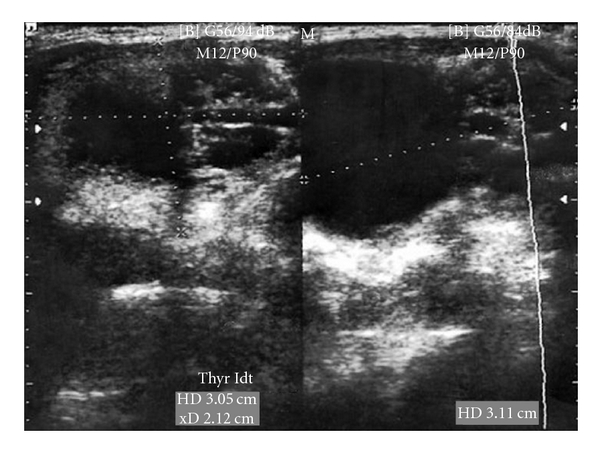
Thyroid ultrasonography showed a large lobulated cystic lesion in the right thyroid lobe and multiples adjacent little cysts in isthmus.

**Figure 2:**
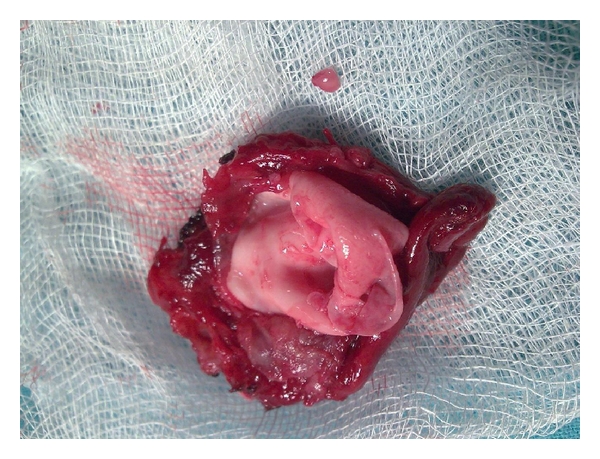
Removed thyroid right lobe and isthmus which includes a 30 mm cystic nodule in the right thyroid lobe contained germinate membrane and daughter vesicles.

**Figure 3:**
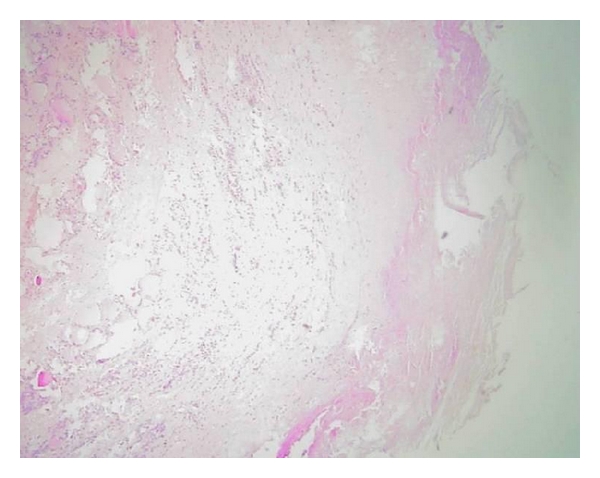
Histopathologic examination of the removed thyroid and cyst. A dense hyalinized fibrous tissue and a mixed inflammatory infiltrate (including eosinophils) were present between the cyst and thyroid tissue. HE×40.

A 23-year-old woman, without any history of farming or raising livestock, presented with an enlarging neck mass, which was noticed 18 months before her presentation. On examination, a well-circumscribed 3.0 × 3.0 cm mass was observed in the right lobe of the thyroid, which was nontender on palpation. No other physical abnormalities were noted. The results of the thyroid function tests as well as routine laboratory tests were normal. An ultrasound study revealed a large lobulated cystic nodule in the right thyroid lobe with multiples adjacent cysts in isthmus (Figure 1). A technetium-99m scan demonstrated that it was a cold nodule. Abdominal ultrasonography and chest X-ray were negative for hydatid cyst. To avoid the spread of protoscolices, fine needle aspirate (FNA) was not performed. The surgery consisted of a subtotal thyroidectomy. We also performed intraoperative ultrasonography to eliminate possible microcysts in adjacent area. No anaphylactic reactions were developed during the operation. Routine wound closure was performed. In gross pathology, there was a 3 cm cystic nodule in the right lobe and multiples cysts in isthmus of the thyroid gland. A thick fibrous wall separated the cysts from the surrounding thyroid parenchyma (Figure 2). Histopathologic examination confirmed the hydatic nature of the thyroid cyst (Figure 3). After the operation, she received Albendazole treatment (400 mg/d) for 2 months. Nonetheless, there was no evidence of any other foci of hydatid disease. At the 24-month followup, a repeated examination showed no recurrence of the hydatid disease.

## 3. Discussion

Hydatid disease is a parasitic infection with worldwide distribution, especially in sheep and cattle-rearing regions of Australia, South America, the Middle East, South Africa, Eastern Europe, and the Mediterranean region [1, 2]. This infection is frequent in Morocco, and the vast majority of our patients live in rural areas [5]. Hydatid cyst of Echinococcus granulosus can be developed in any part of the body. Although it tends mostly to form in the liver (75%) or lung (15%), other organs of the body including brain, heart, bones, muscle, kidney, and pancreas may also be affected [3, 6]. Multiorgan involvement has been reported in 20–30% of hydatid disease cases [3]. 

The parasitic embryo can enter the systemic circulation and lodge in the thyroid gland after either bypassing (primary type) or passing through (secondary type) the hepatic microcirculation [1]. A high blood flow rate in the thyroid gland may be responsible. However, the small diameter of the thyroid arteries explains the rarity of the disease [1, 3]. Hydatid disease caused by Echinococcus granulosus is often manifested by a slow-growing cyst mass. The cyst might remain clinically silent for a long time period [2, 3]. It may suddenly increase in size after years of dormancy [1]. When it increases in size, it may adhere to the surrounding structures, such as trachea, esophagus, carotid sheath, recurrent laryngeal nerve, and the strap muscles, in a similar manner with thyroid carcinoma. Consequently, the patient may present with pressure symptoms and signs such as dyspnea, paralysis of the vocal cord, hoarseness, or dysphagia [1–3]. Complications include anaphylaxis by spontaneous or surgical rupture of the cyst, pyogenic abscess in secondary infected cysts, compression symptoms, and internal organ damage [5]. As described, our patient had symptoms after a long silent period and experienced dysphagia when the cysts grew up considerably. 

Radiologic signs are usually nonspecific. Hydatid origin was suspected in only 50% of patients preoperatively and immunologic testing had a 33% false-positive rate [3, 5]. Diagnosis of hydatid disease has been greatly facilitated with ultrasonography, CT, and magnetic resonance imaging (MRI). The ultrasonography is highly efficient in detecting germinal vesicles in cystic lesions, which is important for a preoperative diagnosis of hydatidosis. Although cystic echinococcosis typically consists of a single unilocular cyst, 20–30% of cases present with multiple cysts in the same or multiple organs [1, 2, 5]. The multiple hypoechogenic images noted within the cystic lesions were thought to represent germinal vesicles of a hydatid cyst [7]. CT-scan and MRI are complementary studies. They provide a precise assessment of the extension into the soft tissues and the calcifications of the peripheral rim of the cyst. The signal from the cysts is inhomogeneous of low intensity on T1-weighted and high intensity on T2-weighted images. T2 measurements do not show significant differences between fertile and sterile cysts [5]. As in our case, thyroid hydatid cyst is reported to present as a solitary cold nodule on thyroid scintigraphy, and may mimic thyroid carcinoma [1]. The routine use of FNA in the workup of a single thyroid nodule may complicate further management of patients with a hydatid cyst by precipitating anaphylaxis and dissemination [1, 6]. Serologic examinations have the problems of low diagnostic sensitivity and specificity, and have only a limited use. HD may mimic benign or malignant tumours, cysts, abscess, and other lesions [3, 5, 7]. 

Management of thyroid hydatid cyst is surgical. Complete excision should be the procedure of choice. The commonly held opinion is that echinococcal cysts should be radically removed whenever possible [1, 5, 6]. The surgeon must be careful to remove the cyst, totally avoiding spilling its contents since fatal anaphylaxis on spilling the contents of the cyst has been reported [8, 9]. Nevertheless, the surgeon should keep in mind that enucleation alone may be associated with local relapse. The plan of cleavage most likely to be entered is that between pseudocyst and ectocyst, and it becomes difficult to avoid rupture of the ectocyst. Moreover, daughter cysts could remain in the adjoining thyroid tissue [1, 2, 8, 9]. We performed subtotal thyroidectomy to serve the purpose. However, the authors also recommend subtotal thyroidectomy especially when the cyst is small and confined to the thyroid gland. Chemotherapy is necessary to avoid recurrence [1–5]. 

In conclusion, hydatid cyst in the thyroid gland is rare. Although inhomogeneous appearance of cystic echinococcosis makes its radiologic diagnosis difficult [9], it should be included in the differential diagnosis of cystic lesions, especially in endemic regions. The definitive diagnosis for most cases of hydatid cyst is possible via such imaging methods. As a result, the surgeon must be aware of the possibility of hydatid disease during the evaluation of thyroid nodules, particularly in endemic regions.
